# Periodic reflections: a method of guided discussions for documenting implementation phenomena

**DOI:** 10.1186/s12874-018-0610-y

**Published:** 2018-11-27

**Authors:** Erin P. Finley, Alexis K. Huynh, Melissa M. Farmer, Bevanne Bean-Mayberry, Tannaz Moin, Sabine M. Oishi, Jessica L. Moreau, Karen E. Dyer, Holly Jordan Lanham, Luci Leykum, Alison B. Hamilton

**Affiliations:** 1 South Texas Veterans Health Care System, San Antonio, Texas USA; 2 UT Health San Antonio, San Antonio, Texas USA; 3 Veterans Affairs Health Services Research and Development Center for the Study of Healthcare Innovation, Implementation & Policy, Los Angeles, California USA; 4 Veterans Affairs Greater Los Angeles Health System, Los Angeles, California USA; 5 David Geffen School of Medicine at University of California, Los Angeles, California USA

**Keywords:** Replicating effective programs, Qualitative methods, Ethnography, Implementation context, Complexity science, Women veterans; adaptation

## Abstract

**Background:**

Ethnography has been proposed as a valuable method for understanding how implementation occurs within dynamic healthcare contexts, yet this method can be time-intensive and challenging to operationalize in pragmatic implementation. The current study describes an ethnographically-informed method of guided discussions developed for use by a multi-project national implementation program.

**Methods:**

The EMPOWER QUERI is conducting three projects to implement innovative care models in VA women’s health for high-priority health concerns – prediabetes, cardiovascular risk, and mental health – utilizing the Replicating Effective Programs (REP) implementation strategy enhanced with stakeholder engagement and complexity science. Drawing on tenets of ethnographic research, we developed a lightly-structured method of guided “periodic reflections” to aid in documenting implementation phenomena over time. Reflections are completed as 30–60 min telephone discussions with implementation team members at monthly or bi-monthly intervals, led by a member of the implementation core. Discussion notes are coded to reflect key domains of interest and emergent themes, and can be analyzed singly or in triangulation with other qualitative and quantitative assessments to inform evaluation and implementation activities.

**Results:**

Thirty structured reflections were completed across the three projects during a 15-month period spanning pre-implementation, implementation, and sustainment activities. Reflections provide detailed, near-real-time information on projects’ dynamic implementation context, including characteristics of implementation settings and changes in the local or national environment, adaptations to the intervention and implementation plan, and implementation team sensemaking and learning. Reflections also provide an opportunity for implementation teams to engage in recurring reflection and problem-solving.

**Conclusions:**

To implement new, complex interventions into dynamic organizations, we must better understand the implementation process as it unfolds in real time. Ethnography is well suited to this task, but few approaches exist to aid in integrating ethnographic insights into implementation research. Periodic reflections show potential as a straightforward and low-burden method for documenting events across the life cycle of an implementation effort.

They offer an effective means for capturing information on context, unfolding process and sensemaking, unexpected events, and diverse viewpoints, illustrating their value for use as part of an ethnographically-minded implementation approach.

**Trial registration:**

The two implementation research studies described in this article have been registered as required: Facilitating Cardiovascular Risk Screening and Risk Reduction in Women Veterans (NCT02991534); and Implementation of Tailored Collaborative Care for Women Veterans (NCT02950961).

## Background

Implementation science was founded on the recognition that achieving uptake of evidence-based practices can be challenging for a variety of reasons, beginning with the fact that evidence-based practices themselves are typically complex cultural products [1]. Even introducing a relatively simple clinical practice may require multiple steps, integration of new knowledge, and coordination across a chain of individuals. Healthcare personnel responsible for implementing new practices must integrate new practices with existing knowledge, beliefs, and practices [2, 3]; more often than not, they must achieve this integration in dialogue with diverse social partners, including patients, staff, other providers, and leadership [4]. Healthcare settings themselves vary greatly in size, scope, and populations served, and are inherently multi-level and dynamic, providing a context and ecology into which any new intervention must fit. In light of these challenges, achieving uptake of evidence-based practices is almost inevitably a complex undertaking [5].

One response to growing recognition of this complexity has been the call for greater precision in defining, specifying, and evaluating implementation strategies used in change efforts [6–8]. Proctor et al. [7] have provided guidelines for careful documentation of implementation strategies in order to identify which strategies are most effective in support of implementation efforts, and how those strategies must be operationalized in order to achieve maximum results. More recent work has provided a compilation of implementation strategies to draw upon [6] and innovative examples of how to achieve careful description of implementation strategies in implementation research [9]. Although the need to provide robust description of a planned intervention is well-established following several decades of clinical trials, there is increasing recognition that interventions too – the very evidence-based practices we are trying to implement – have a tendency to evolve as they move into routine practice [10, 11]. Stirman et al. [12] offer a useful taxonomy of common ways interventions are modified as they are put into practice. Innovative theoretical models within implementation science, including the Context and Implementation of Complex Interventions framework [13] and Dynamic Sustainability Framework [10], urge an appreciation for how both an intervention and the plan for its implementation may evolve over the course of implementation, and how implementation may be affected by shifting local or national context. Few recommendations have been put forward, however, regarding how to achieve rigorous specification of intervention and implementation strategies while simultaneously accounting for dynamic ecology. The answer clearly lies in careful observation and documentation as part of implementation research [7, 9], but few methods have been proposed to address this challenge, particularly in the context of multi-site implementation trials.

Ethnography is an iterative, flexible methodological approach characterized by close engagement with a social group over time in a manner that “permits access to people’s social meanings and activities” [14: 312]. Although ethnographic methods often include participant observation, this is not necessarily the case, as they also include a variety of techniques including in-depth interviews, discourse analysis, and review of documents or vignettes, making use of multiple methods to ensure triangulation of data sources and allow “thick description” of setting and events [14–16]. Ethnography is also associated, as John Brewer has written, with a naturalistic philosophical framework that aims to understand “people’s actions and experiences of the world, and the ways in which their motivated actions arise from and reflect back on those experiences” [14: 313].

Ethnographic methods have been recommended for use in implementation and process evaluations [15, 17–19] and are well-suited to observations of events, relationships, and context over time [20]. Because ethnographers engage with and observe participants in a prolonged way, as events are occurring, and in naturalistic settings, ethnography produces data of high validity, helping to avoid common research pitfalls related to social desirability or post-hoc explanations offered long periods after events have occurred [18]. Despite its many benefits, ethnography can present challenges in pragmatic research. It can be time-intensive during data collection and analysis, and as a result, costly [18]; it may therefore be impractical for use in multi-site studies or as part of unfunded or quality improvement projects.

Resource intensity notwithstanding, ethnography may have underappreciated benefits for implementation research. Ethnography emphasizes thoughtful, relatively unstructured discussions of events, engaged in over time with multiple actors in a given social setting. Ethnographic methods can therefore allow for ongoing discussions of implementation phenomena, including features of the implementation context or descriptions of how actors are making sense of events as they occur. In addition to their value for documentation, these types of discussions may also provide valuable space for implementation team members to engage in the critical reflection that can facilitate problem-solving [17, 21–23]. Implementation teams must be responsive to the surrounding ecology and able to adapt as needed, often quite rapidly. Problem-solving within complex settings relies upon effective sensemaking, a group process that enables people to make sense of events as they unfold and to develop real-time insights and solutions [21, 22, 24, 25]. Lanham et al. [21, 26] have illustrated how sensemaking and learning are supported by strong interpersonal relationships and the availability of time and space for reflection. Even so, it can be unusual to take time for non-action-oriented discussion amid the time and funding constraints of research and implementation activities. Regular team meetings, for example, are common in research, but may be task-oriented and directive rather than descriptive and reflective. At least two studies in recent years have offered strategies to enhance team sensemaking using semi-structured discussion tools [27, 28]. Guided reflection as a sensemaking activity within the implementation team merits deeper exploration as an ethnographically-informed approach to understanding dynamic implementation phenomena.

In 2015, the Department of Veterans Affairs (VA) Quality Enhancement and Research Initiative (QUERI) funded a five-year, multi-site program of research aimed at “Enhancing Mental and Physical Health of Women through Engagement and Retention” (EMPOWER) [29]. EMPOWER includes three projects to implement innovative care models in VA women’s health using Replicating Effective Programs (REP), an evidence-based implementation strategy [30–32] enhanced with stakeholder engagement [33] and complexity science [13, 21, 34, 35]. As part of a multi-method assessment strategy, we developed a pragmatic, ethnographically-informed method for guided discussions (“periodic reflections”) to be used across EMPOWER. Periodic reflections aid in documenting and encouraging reflection on key implementation events, actors, and processes, including adaptation, in complex, multi-site, multi-level implementation studies. This paper has three primary goals: (1) to describe periodic reflections as a method for guided discussions and how they have been used as part of EMPOWER’s implementation evaluation; (2) to illustrate, using examples from all three EMPOWER projects, the value of periodic reflections as a low-burden method for capturing time-sensitive data of interest in implementation trials, and for helping to operationalize dynamic context, adaptation, and team sensemaking in complex interventions and settings; and (3) to consider how periodic reflections may also support effective sensemaking and learning within implementation teams.

## Methods

### Description of the EMPOWER QUERI

EMPOWER is comprised of three projects sharing an implementation strategy and core methodological approach [29]. The first of these projects, a quality improvement (QI) project, entitled “Tailoring VA’s Diabetes Prevention Program to Women Veterans’ Needs,” has been completed in VA Greater Los Angeles women’s health clinics. Women Veterans with prediabetes were invited to participate in a gender-specific, evidence-based diabetes prevention program (DPP) [36–38] to support healthy lifestyle change, and were presented with the option of either in-person, peer-led or online, professionally-moderated DPP groups. The other two EMPOWER projects are four-year research studies occurring across multiple sites. The first of these projects, “Facilitating Cardiovascular Risk Screening and Risk Reduction in Women Veterans” (known as CV Toolkit), aims to reduce CV risk among women Veterans by increasing identification of and enhancing patient/provider communication around CV risk, and by providing a coaching intervention to support women Veterans’ engagement and retention in appropriate health services through a facilitated women-only group intervention. The final project, entitled “Implementation of Tailored Collaborative Care for Women Veterans” (CCWV), is evaluating implementation of an evidence-based collaborative care model for women Veterans with anxiety, depression, and/or PTSD treatment needs, toward the goals of improving the effectiveness of primary care-mental health integration (PC-MHI) and women Veterans’ engagement and retention in PC-MHI.

**Fig. 1:**
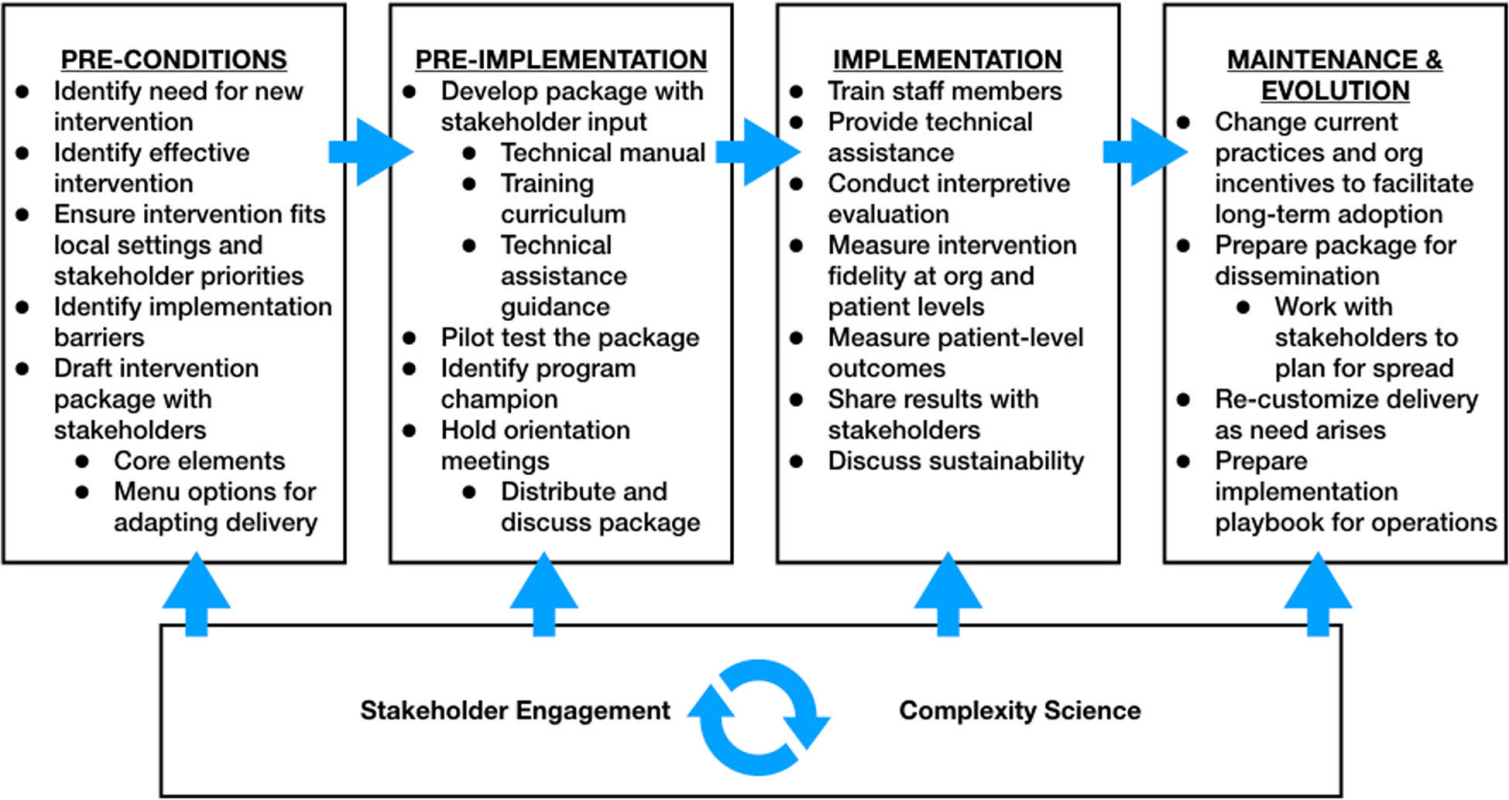
Replicating Effective Programs (REP) Implementation Strategy Enhanced with Stakeholder Engagement and Complexity Science (Adapted from [30, 75])

REP, the implementation strategy used across EMPOWER, was developed to support dissemination of evidence-based practices in low-resource settings [32], and has since been used widely to facilitate implementation of a variety of interventions [30, 31]. REP offers guidance for adapting existing interventions for use in novel settings or with new populations, describing a sequence of activities (e.g., needs assessment; see Fig. 1) occurring across four phases: pre-conditions, pre-implementation, implementation, and maintenance and evolution [30]. Use of REP across the three EMPOWER projects allows for tailoring existing evidence-based interventions to meet the needs of women Veterans in VA primary care, with adaptation occurring in real time across multiple sites.

VA has struggled to meet the needs of its rapidly growing population of women Veterans, who experience persistent disparities in control of risk factors for diabetes and CV conditions [39, 40], high rates of depression and anxiety [41], and organizational barriers to VA care (e.g., lack of gender-specific services) [42–45] resulting in high attrition [46, 47]. In order to better meet the needs of women Veterans in VA primary care, we enhanced REP by integrating multi-level stakeholder engagement [33] and complexity science [48] throughout, examining VA health care as a complex adaptive system [48]. Complex adaptive systems are characterized by a large number of interconnected but diverse agents who engage in self-organization and co-evolve within a complex and dynamic environment; as a result of this diversity and continual evolution, events within complex adaptive systems may be unpredictable and their impacts nonlinear [21, 27]. By augmenting REP with stakeholder engagement and complexity science (see Fig. 1), our enhanced implementation strategy supports continual engagement with participants across all levels of VA’s healthcare organization, including the women Veterans it serves, while inviting attention to ways in which the intervention, use of implementation strategies, and context shift over time.

To operationalize this conceptual approach and implementation strategy across three projects occurring at multiple sites, we developed a mixed-method plan to support process evaluation and inform meaningful understanding of results (Table 1). Each of the three EMPOWER projects makes use of a similar set of qualitative and quantitative methods, including patient and provider surveys, patient and stakeholder interviews, examination of administrative data, and text analysis of study documentation [49]. Following the lead of prior studies using structured templates to guide discussions or written reports as part of ethnographically-informed implementation evaluation [15, 27, 50], we developed a preliminary template for guided discussions, tailored for use in telephone conversations occurring at monthly or bi-monthly intervals across the EMPOWER projects. The initial “periodic reflections” template was revised according to feedback from a series of experts in implementation and qualitative research, including members of our Strategic Advisory Group, and regular reflections were conducted beginning in June 2016. The template has been iteratively refined over time to ensure compatibility with EMPOWER project needs and goals, as described below.

**Table 1:** EMPOWER QUERI Implementation Evaluation: Summary of Methods (Reprinted with permission from Huynh et al. [76])

*Replicating Effective Programs (REP) Phase*	PHASE 1 Pre-Conditions						PHASE 2 Pre-Implementation						PHASE 3 Implementation															Phase 4 Maintenance & Evolution			
*Month*	1	2	3	4	5	6	1	2	3	4	5	6	1	2	3	4	5	6	7	8	9	10	11	12	13	14	15	1	2	3	4
Provider and Administrator Interviews • Phase 1: Intervention planning, needs assessment, and acceptability; • Phase 2: Factors likely to affect adoption, acceptability, feasibility, satisfaction, penetration/reach. • Phase 4: Experiences of intervention/implementation; adaptations made in practice; suggestion for future adaptations to inform effectiveness and spread.				X						X																		X			
Provider Surveys • Measuring Organizational Readiness for Patient Engagement (MORE)										X																					
Patient Interviews • Phase 3: Factors likely to affect adoption, acceptability, feasibility, satisfaction, penetration/reach. • Phase 4: Experiences of intervention/implementation; challenges, problem-solving, and suggestions for change/adaptation.													X															X			
Patient Surveys *(pre and post-intervention)* • Primary outcomes: Program engagement and retention; change in targeted symptom or risk reduction behavior; • Secondary outcomes: Satisfaction (at f/u only), global health, out of role days; • Potential moderators: engagement, patient demographics, social support, mental health.													X												X						
Periodic Reflections • History and trajectory of implementation events • Activities and interrelationships, including stakeholder engagement; • Adaptations to intervention components and/or implementation strategies; • Contextual factors with potential impact for implementation.							X	X	X	X	X	X	X	X	X	X	X	X	X	X	X	X	X	X	X	X	X	X	X	X	X
Administrative Data • Referral monitoring • Patient engagement • Patient outcomes												X	X	X	X	X	X	X	X	X	X	X	X	X	X	X	X				
Text Analysis • Review of changes occurring to intervention components and/or implementation strategies per T1 (Baseline) proposal materials and subsequent institutional review, amendments, and other study documentation.																												X			

### The method and content of periodic reflections

Table 2 provides description of the “periodic reflections” guided discussions template, including the rationale and guidance for each component. The primary purpose of periodic reflections is to ensure consistent documentation of key activities and other phenomena (e.g., challenges, adaptations, etc.) occurring over the course of implementation. Reflections are completed as 30–60 min guided telephone discussions with multiple members of the implementation team, including project PIs, site leads, and/or other team members as appropriate to the phase of the study and main focus of current activities. Reflections are facilitated by a member of the EMPOWER Implementation Core (“reflections lead”), a PhD-level anthropologist, who documents discussion content in detailed notes. On a month-to-month basis, we seek participation from individuals whose roles are most likely to expose them to novel information regarding implementation phenomena in a given period. For example, PIs are often in a position to provide information regarding both day-to-day activities and ongoing negotiations with site leadership or national partners, and thus are frequent participants in periodic reflections. However, we have also found it valuable to include site-based staff (e.g., site PIs, project managers), particularly following the introduction of new activities (e.g., implementation launch) or when challenges have arisen (e.g., low rates of patient participation in an early coaching group). Reflections are project-specific (i.e., limited to DPP, CV Toolkit, or CCWV teams). Although calls are flexible and have included up to five individuals, including the reflections lead, individuals fulfilling different implementation roles ordinarily participate in separate reflections to support gathering diverse perspectives. Reflections are routinely scheduled at monthly or bi-monthly intervals. The amount of time between discussions is dependent upon the level of current study activity, with wider spacing during periods of more routine implementation.

**Table 2:** “Periodic Reflections” Guided Discussions Template

Main Components	Rationale and Guidance
Introduction * Goals and Focus: These reflections are intended to provide an opportunity to check in regularly about how implementation efforts are going. Our main goal is to take a few minutes to discuss, document, and reflect on key activities, events, and changes occurring over the course of implementation.*	Sets stage for core goals of observing, documenting, and reflecting on implementation-related events and phenomena. Serves as instructive language during early reflections, helping participants become accustomed to the process. In later sessions, provides an orienting reminder of the goals of the activity.
Date Completed by reflection lead	Allows linkage to implementation phase, events. Periodic reflections provide a means to gather repeated, consecutive information regarding implementation events and conditions occurring at specific moments over the course of an implementation effort. Data can be reviewed retrospectively to reveal changing phenomena and/or sensemaking over time, and, in longitudinal analyses, can aid in understanding fluctuations in implementation or clinical outcomes.
Participant Names/Roles Completed by reflection lead	Provides information on the role of participating team members. Key agents may vary across time according to changing teams or study needs, implementation phase, or site involvement.
Status update * What are the current main activities for the project? How is it going?*	Open-ended invitation to discuss the implementation project generally, including major activities and current sense of challenges and successes. Prompts may be used as needed to encourage discussion of day-to-day efforts, recent accomplishments and completed tasks, as well as barriers that have arisen and the sensemaking and problem-solving that has occurred in response. Open discussions may help the group to strengthen connection and gain new insights on recent events. When multiple team members are participating, open dialogue and turn-taking is encouraged.
Adaptations to Intervention * Have there been any changes to how the intervention is delivered in the past month or so?*	Observing, documenting, and reflecting on adaptations to the intervention aids in understanding mechanisms and outcomes of program impact.
Adaptations to Implementation Plan * Have there been any changes to the implementation plan in the past month or so?*	Observing, documenting, and reflecting on adaptations to the implementation plan, with value for understanding what implementation strategies were undertaken and how agents responded. Aids in refining plans for scale-up and spread.
Stakeholder Engagement * Have there been any stakeholder engagement efforts in the past month?*	Tracking of specific outreach efforts made in service of research or implementation efforts; provides an opportunity to capture formal and informal activities aimed at supporting interdependencies with local and national partners.
Environment/Context * Have you seen any recent changes in the local or national environment that you think may have impact for implementation?*	Acknowledges the unpredictability of implementation settings, as well as how changing conditions across multiple levels (local, regional, national) can impact the success of implementation efforts. Prompts continued attention to contextual conditions, supporting opportunities for study documentation, novel sensemaking, and/or adaptation as needed.
Planning * What are the next steps going forward?*	Provides opportunity for discussing expected activities over the coming weeks, helping to link discussion of recent events and conditions to plans for future action.
Additional Prompts (for use as needed) • *Have particular barriers/concerns have arisen recently? What solutions have been tried? How is that going?* • *Who have been the key people involved in recent activities, efforts, and discussions? What have been their primary concerns, hopes, and/or suggestions?* • *Have there been any surprises lately, or unexpected events?* • *What lessons have been learned?*	

Unlike more formal structured or semi-structured interview guides [51], the guided discussions template functions as a lightly-structured invitation for members of the implementation team to attend to, discuss, and document ongoing activities and implementation phenomena, including recent challenges and problem-solving efforts, changing features of the local or national context, and adaptations to the intervention or implementation plan. Implementation science has seen increasing attention to the near-inevitability of adaptation as interventions are disseminated into new settings and adopted by new practitioners, and as agents within the implementation effort respond to changing conditions, including the transition from initial implementation to sustainment [52]. Similarly, adaptations may be made to the planned rollout of implementation strategies as knowledge of the setting increases and conditions of the site, relationships, and/or context evolve.

Researchers have proposed a variety of novel methods for observing [49] and documenting such adaptations [53], including pre-populated tracking logs completed by study personnel. We have taken an alternate approach by asking participants to describe recent changes to the intervention or implementation plan, and by probing, where necessary, for discussion of why and how such changes were introduced. This more open-ended approach was selected for two reasons. First, there is considerable flexibility to this method, which requires no front-end delineation of expected changes. This was of particular value in EMPOWER, given shared methods across projects of diverse types and components, which include DPP lifestyle change groups, provider use of electronic medical record-based templates for CV risk reduction, and primary-care-based care management for mental health. Second, agents involved in implementation, including members of the implementation team, may not always recognize when their actions reflect a change from an intended practice or protocol [12, 49]. Although guided discussions cannot provide the same granularity regarding intentional and unintentional modifications as might direct observation, we thought it likely that the reflective quality of the discussions would provide a window onto activities through which unrecognized adaptations might be observed. Table 2 provides additional detail on questions related to adaptation in the template.

In facilitating periodic reflections, as in some other forms of qualitative research [54], we have found it helpful for the reflections lead to walk a balance between the naïve interviewer, whose knowledge of events and conditions may be limited, and the insider, who is expected to have a reasonable understanding of background and current events. Follow-up questions are frequently necessary in order to clarify information for documentation purposes. Because of the frequency of reflections over the course of implementation, there is the luxury of time to develop trusting relationships with participants, which supports valid data collection. This trust and the resulting quality of the reflections as a data resource are two of the most significant strengths of this method. The recurrent nature of periodic reflections also provides the opportunity to follow up on topics raised in prior discussions to reveal shifts in conditions or sensemaking.

### Analysis

As noted above, periodic reflections are one component of EMPOWER’s multi-method evaluation plan (summarized in Table 1). In alignment with our use of an enhanced REP strategy, study methods combine qualitative and quantitative data collection strategies to accomplish four research- and implementation-focused aims: namely, to: (1) support iterative tailoring and adaptation of EMPOWER interventions over the phases of implementation; (2) provide data on factors affecting implementation outcomes (i.e., adoption, acceptability, feasibility, satisfaction, and penetration/reach) [55], from the perspectives of key stakeholders including leadership, providers, and patients; (3) provide data to evaluate implementation and patient outcomes associated with each project; and (4) inform development of intervention packages, including refined implementation playbooks [56], for dissemination during scale-up and spread. Data from periodic reflections can be examined alone or in triangulation with other data sources in support of each of these aims [57]. For example, data from baseline provider interviews have been integrated with reflections occurring during implementation at an initial site to inform tailoring of communication strategies prior to launch at later sites. Similarly, findings from post-implementation interviews are being integrated with administrative data on intervention uptake and contextual data drawn from reflections to better understand factors impacting implementation adoption across sites.

We have taken an ethnographic approach to analysis, conducting continual review and coding of reflections and other qualitative data (e.g., patient interviews conducted pre- and post-DPP participation) in order to inform evaluation and implementation activities. One benefit of the reflections method is the flexibility of the template, which can be iteratively refined to align with changing study needs over time. In the same way, the resulting data can be analyzed using a variety of approaches, to meet formative or summative needs, and in real time and/or retrospectively. Reflections data are appropriate for use with multiple analytic approaches, including matrix [58] or rapid qualitative [59] analyses. Because we do not audiorecord the reflections, relying instead on detailed notes taken by the call lead, we do not consider the resulting documents appropriate for more granular discourse or content analysis; those interested in making use of such techniques could audiorecord the guided discussions, with the tradeoff that transcription would be optimal.

Coding of periodic reflections to date has been conducted in ATLAS.ti [60] using a strategy that begins with coding sequences of text for broad concepts defined a priori. In accordance with our use of REP enhanced with complexity science as an implementation strategy, we have an analytic focus on dynamic context, adaptations to the intervention and implementation plan, and team sensemaking and learning; we therefore conducted an initial round of coding to identify text relevant to these phenomena. Results of these preliminary analyses are described below. Additional analysis is ongoing. For example, we are further coding references to adaptation according to existing frameworks, with modifications to the intervention coded per the Stirman taxonomy [12] and references to implementation strategies coded using a combination of the Powell et al. [6] compilation (where new implementation strategies were introduced) and Proctor et al. [7] guidelines for specification (where elements of an implementation strategies were modified while retaining use of the core strategy). As coding has proceeded, we have also identified emergent themes and codes in the data, such as the impact of unexpected events in implementation. Inductive codes identifying these phenomena have been integrated into the codebook for systematic use.

At the current time, data collection remain ongoing across all three EMPOWER implementation projects. DPP, which has the shortest time frame, is the sole project to be in the final stages of implementation.

## Results

Four EMPOWER teams, including three core project teams and one site-based team, completed 30 periodic reflections over the 15-month period between June 2016 and September 2017; initial reflections were conducted during the pre-implementation phase for all projects. Table 3 provides information on the frequency of reflections occurring across projects, the role(s) of participants, and implementation phases covered. When conducted monthly, periodic reflections require approximately 70–90 min per team per month, inclusive of scheduling, discussions, finalizing notes, and data management. In the following paragraphs, we examine how reflections data have facilitated documentation of implementation phenomena related to dynamic context, adaptations to the intervention and implementation plan, and team sensemaking and learning across the three EMPOWER projects (see Table 4 for additional examples).

**Table 3:** Characteristics of EMPOWER Periodic Reflections Data

EMPOWER Study	Number Completed (*n* = 30)	Implementation Phases	Participants
Tailored Diabetes Prevention Program (DPP) for Women Veterans	9	Pre-Implementation, Implementation, Maintenance and Evaluation	PI, Team Coaches
Cardiovascular Risk Screening and Reduction for Women Veterans (CV Toolkit)	13	Pre-Implementation, Implementation	Co-PIs, Project Coordinator
Collaborative Care for Women Veterans (CCWV)	8	Pre-Implementation, Implementation	PI, Co-PI, Co-I, Site Leads, Site-based Staff

**Table 4:** Examples from Periodic Reflections Across EMPOWER Projects

Sample Domains	Examples
Dynamic Implementation Ecology	• CCWV: Characteristics of the local site
	*“[Site] is an amazing site. I don’t think it’s inconsequential that they have such a strong PACT leader and mental health leader, and stable leaders as well, and champions in the field.* *Doing really innovative things, and dedicated to quality improvement…they have a lot of good stuff going on.” [Study Lead, Pre-implementation Phase]*
	• DPP: Shifting national policy environment
	*“[There’s change in] the climate around doing remote delivery of healthcare, which I think VA is increasingly interested in….different than two years ago when we submitted the [project grant] proposal.” [Study Lead, Maintenance Phase]*
Adaptations to the Intervention	• CV Toolkit: Adding a co-facilitator for *Gateway for Healthy Living* groups.
	*“One of the…recommendations that we were going to incorporate…The report said that facilitators liked when they co-facilitate with someone else – things seemed to run better. So we want every site to have a co-facilitator and probably a back-up so they could run sessions with two people or also with one.” [Study Co-Lead, Pre-implementation Phase]*
	• CCWV: Expansion of care manager role.
	*“…[W]e’re going to find it useful for [care manager] to have a little bit broader responsibility than we imagined. She won’t get perfect referrals, but doing the triage ourselves will be better than trying to get the primary care team to do it.” [Study Co-Lead, Pre-Implementation Phase]*
Adaptations to the Implementation Plan	• DPP: Expansion of the program beyond initial plan.
	*“We have decided to send out another few hundred invitations because we have the capacity.” [Study Lead, Pre-Implementation Phase]*
	• CV Toolkit: Addition of a patient-facing communications plan.
	*“The other thing that developed…was that we ended up having to have a communication plan….The marketing strategy.” [Study Co-Lead, Implementation Phase]*
Team Sensemaking and Learning	• DPP: Sensemaking around an appropriate space for in-person groups.
	*“The room we’d planned to use isn’t conducive because of the chairs and tables. We worked with [clinic leads] to find a space next to the clinic. We didn’t want women to have to go too far where they might be subject to harassment.” [Study Lead, Implementation Phase]*
	• CV Toolkit: Learning the importance of an on-site clinical partner.
	*“In terms of lessons learned, the most important thing that happened was [the on-site clinical partner] showing up. The moment [she] walked in, everything changed….I didn’t know she was going to have such an impact on the clinical side.” [Study Co-Lead, Pre-implementation Phase]*

### Dynamic implementation ecology

Reflections data demonstrate two ecological phenomena of central interest for implementation: (1) characteristics of the local, regional, or national context that may impact implementation or sustainment, and (2) changes within the implementation environment occurring over time. In one case, a CCWV team member described how the PC-MHI collaborative care model to be implemented aligned well with the existing organization of care at a particular site:

*There was already a role [at the site], so we’re just plugging one more person into…it’s just an extension of what’s already there. Which means I’m going to be kind of surprised if this isn’t easy, if people don’t just go, ‘oh, another care manager, but this one’s for women’…[Site Lead, Pre-implementation Phase]*

Reflections data proved equally useful in capturing dynamic conditions at multiple levels. CV Toolkit, for example, encountered a sudden increase in staffing pressure at one site not long before implementation:

*“So, on the [site name] site we lost our women’s health psychiatrist and then there was a cross-covering psychiatrist covering the first two months of the year, and then she turned in her resignation….She’s still there but they’re expecting it to go crazy [when she leaves].” [Study Co-Lead, Pre-implementation Phase]*

Shifts in the national policy environment were also apparent in these data, as when, midway through DPP implementation, the Center for Medicare and Medicaid Services announced they would begin covering DPP as a benefit within the coming year, prompting reassessment of expectations for scale-up. By supporting documentation of the shifting conditions for implementation for each project, we expect these data to be of value in understanding implementation outcomes and adaptive planning for sustainment and spread.

### Adaptations to the intervention

One of EMPOWER QUERI’s central goals is to support tailoring and adaptation of existing evidence-based practices to better meet the needs of women Veterans in VA primary care. Periodic reflections have provided insight into the adaptations made as implementation progressed, as well as the rationale for these changes. For example, the DPP implementation team modified the original plan for peer-led in-person groups, which normally includes 22 sessions over 12 months, to add a monthly maintenance session continuing after weekly meetings have concluded. Reflection notes from the initial discussion of this option read as follows:

*The professional coach…said last week, “I could just cry – how wonderful the sharing between the women in the groups is …It’s pretty amazing the relationships that have been building the last couple of months. What are we going to do when this finishes? I hope they will have an opportunity to meet.” In the past we’ve done maintenance groups once a month or something and we can do that if enough people are interested. [Study Lead, Implementation Phase]*

Some months later, the issue was raised again:

*Some women have asked [the peer coach] to do a monthly maintenance class, who were really gung-ho, and we have salary support through September, so an hour a month is fine. It’s a very small number [of interested women]. [Study Lead, Implementation Phase]*

In this case, the reflections not only documented the adaptation and its timing (first considered mid-way through implementation, decided upon as the implementation phase was nearing completion), but also captured some of the factors considered by the implementation team in making the decision, including the unexpected closeness developed among women in the in-person groups, the number of participants likely to be interested in additional sessions, and availability of funding to cover the additional service.

### Adaptations to the implementation plan

Likewise, reflections data provide insight regarding shifts in the implementation plan, as when the CCWV project moved from a group-oriented to a one-on-one training model for its site-based care manager position:

*…[W]e were originally going to have [trainer] and her team come and train [care managers from all sites] at the same time, but because of the way things are rolling out, it’s not going to be able to happen all at once. [Study Co-Lead, Pre-implementation Phase]*

In making this decision, the CCWV team was responding to the fact that not all sites were able to launch implementation at the same time. Developing a more flexible, individualized training plan provided the added benefit of allowing more tailored training to meet site needs.

### Team sensemaking and learning

Periodic reflections also reveal team sensemaking as team members responded to new information emerging over the course of the implementation effort. For example, the DPP team engaged in thoughtful reflection around observations of the women’s in-person groups that impacted how they viewed mechanisms of action for the intervention:

*We can’t change someone’s financial [situation]. Women [in DPP groups] are giving each other advice on where to buy fresh produce. It’s trying to get at the issues that are probably why these women are so obese and have health issues to begin with. No class is going to teach these things. The real life translating to your real-world situation. I don’t know what the family and social issues are, but there’s a lot of talk about that. Those are the things that can motivate or really unmotivate somebody. [Study Lead, Implementation Phase]*

This quote illustrates the implementation team’s emerging view that the “active ingredient” of this intervention is not only the education on healthy lifestyle provided in the groups, but also the stories and support shared among women participants regarding how to achieve positive lifestyle change amid ongoing life challenges.

Reflections data also reveal much about how learning has occurred over the course of implementation. As implementation proceeds, conditions shift, and/or challenges arise, team members come to new understandings around what is happening and how best to move forward. As an example, in-person DPP groups were run by a peer leader named Alyssa (pseudonym). Alyssa is a woman Veteran who had herself participated in an earlier DPP group and been successful in losing weight; she proved to be more effective in engaging with group members than a prior peer leader who had been through the same training. A DPP team member noted at the time:

*We tried for six months to train [the prior leader] and [Alyssa], and [the prior leader] did a good job, but just because somebody’s a Veteran isn’t going to make them good at this…The fact that [Alyssa] has prediabetes and was able to make the changes, lose the 40 [pounds], [Alyssa] is a walking testament to the program. [Study Lead, Maintenance and Evolution Phase]*

Reflections data reveal how the team began to consider that being a successful DPP peer leader requires more than being a Veteran. In doing so, they took steps to refine expected role requirements for a successful DPP peer leader, integrating this information into planning for scale-up and spread.

Finally, although initially intended primarily as a recurring strategy for documentation, periodic reflections also appear to function as an activity that itself supports connection, sensemaking and learning within the implementation team. One CV Toolkit team member noted that reflections had become *“integral to understanding what we are doing and how it is going, flowing, or getting stuck, or not starting.*” As another team member put it:

*“If I understand sensemaking correctly, then I could see it being one of the primary benefits of periodic reflections. We never take time in usual projects to just talk about what has happened and what we should do later. Reflections make us do that.” [Study Co-Lead, Implementation Phase]*

## Discussion

Responding to a relative lack of consensus regarding how to achieve adequate documentation of dynamic implementation phenomena, this paper describes periodic reflections as a method used within EMPOWER’s implementation evaluation and offers some illustration of how reflections data are helping to observe dynamic implementation context, emerging adaptations, and team sensemaking across the EMPOWER projects. Our experience to date indicates that inclusion of periodic reflections as part of a multi-component evaluation strategy is contributing to a comprehensive picture of how EMPOWER projects are evolving in real time. Periodic reflections represent a straightforward and low-burden method that provides rich data on the life cycle of an implementation effort, informing both real-time and retrospective analyses. Reflections feature some of the strengths of an ethnographic approach, including close engagement and relationships with active participants in the process and a method that can be adapted to meet changing study needs [15, 17, 18]. Included as part of a multi-method study design, these guided discussions may offer a pragmatic way to gather ethnographic insights in real-world implementation research.

The contribution of an ethnographic approach is also salient with regard to the timing of data collection. Although qualitative methods are a common feature in implementation studies [20, 38, 61, 62], they have typically been used in a punctuated fashion, occurring at pre-, mid-, or post-implementation. Only in recent years has continuous use of qualitative methods across implementation begun to be seen more frequently [15, 50]. It is widely recognized that data gathered months or years following key events brings with it risk of recall bias and retains diminishing validity [63]; by contrast, periodic reflections situated in the time and context of ongoing implementation produce data that are nuanced, detailed, and illustrative of change as events progress.

Our use of the reflections method provides one example of how an ethnographic mindset can be applied to understanding complex phenomena in implementation research, but many others exist. Prior studies, for example, have treated study documents such as regulatory approvals or notes from facilitation or team-based coaching as data sources to support ethnographic analysis [20, 49]. In a recent article, Bunce et al. [15] described how taking an ethnographic approach within implementation and evaluation research “emphasizes placing the intervention in its historical and social context, ‘being there’ to document the process as it unfolds and as interpreted by its participants, openness to unanticipated consequences, and illumination of multiple, complex, and competing perspectives” (pg 15). Although periodic reflections conducted over the phone lack the detail of ‘being there’ in-person, and cannot replace the observation component of classic ethnography [51], they offer an effective means for capturing information on context, unfolding process and sensemaking, unexpected events, and diverse viewpoints, illustrating their value for use as part of an ethnographically-minded implementation approach.

We have found that these guided discussions support effective documentation of specific events, such as adaptations to the intervention or implementation plan, while also capturing the dynamic interplay of other phenomena that impact implementation success, including use of implementation strategies, aspects of the setting and/or policy environment, and team sensemaking [27]. The resulting data can inform implementation evaluation, while the data collection itself provides an opportunity to reflect on implementation successes, challenges, needs, and opportunities as they arise. Periodic reflections thus appear to have a dual function: initiated as a method for rigorous documentation of implementation activities and phenomena, they also have benefit in supporting effective team sensemaking and problem-solving. Documentation as conducted for EMPOWER was intended as an evaluative or research-focused activity; however, reflection has also emerged as a sensemaking activity that iteratively informs how both research and implementation activities are understood and conducted. This is perhaps not surprising, as encouraging reflection on problems, gaps, and ways of working is increasingly common across implementation strategies, including mentored implementation [64], reflective adaptation [65], and implementation facilitation [66–68]. Facilitation itself is thought to be based in interactive problem solving, relationship building and effective communication [66, 68], all of which may be supported by the action of regularly taking time to reflect on how implementation is proceeding. Design principles for encouraging sensemaking in organizations similarly encourage providing opportunities for interactive communication and “noticing and bracketing” information for further interpretation, towards the generation of a “plausible story” to aid in assessing the need for further action [25, 69, 70]. It may be that periodic reflections, by facilitating timely identification of needed modifications or adaptations, can help to avoid or reduce ineffective use of resources by supporting teams in identifying problems at an earlier stage [23]. The value of reflections as an ethnographic method may further increase where reflections function to actively support implementation, effectively positioning the reflections lead as a participant-observer within implementation itself. With this is mind, we are continuing to examine how periodic reflections inform the conduct of implementation as the EMPOWER studies proceed.

We have also found this ethnographically-informed method to be highly compatible with our use of an enhanced REP implementation strategy. Like many strategies based in iterative or participatory research, REP relies upon formalized cycles of reflection and action [71, 72], and the periodic reflections described here provide rich, recurring data to complement data collection activities occurring at rarer time points (e.g., pre- and post-implementation). In addition, we have found reflections to be a useful tool for operationalizing complexity science in implementation, particularly in demonstrating the evolving sensemaking of actors over time and in relation to the shifting dynamics of the implementation itself. Given the flexibility of the periodic reflections method, we anticipate it to be of value for use with a variety of implementation strategies and conceptual approaches.

Although we have found periodic reflections to be a convenient and worthwhile strategy for data collection, there are limitations associated with this method. Completing reflections on a regular basis requires commitment and buy-in from project teams, who must agree to participate on a recurring basis. The guided discussions are most effective in the context of strong trust developed between the reflections lead and implementation team members [26, 27]. Although the regularity of the reflections provides a natural opportunity to develop trusting relationships, the reflections themselves are unlikely to be successful where implementation team members lack a feeling of psychological safety in describing problems and missteps as well as successes. Periodic reflections may not provide opportunity to observe the differences between what people say and what they do, or to observe phenomena (e.g., conflicts) that are not described by participants [51], and thus cannot replace the detailed information available via in-person observation.

It is worth noting that the reflections template as tailored for EMPOWER does not provide easily quantifiable data in aid of evaluation and assessment – e.g., regarding the number of hours engaged in specific implementation activities over the course of a given month. However, the method is sufficiently flexible to be adapted to meet a variety of study needs. There is no reason why more quantitatively-focused questions could not be included, as is common in formalized logs for tracking use of implementation strategies [9], with the caveat that creating an overly structured template may inhibit the open and reflective dialogue that is a primary benefit of this method.

Finally, periodic reflections are in current use as part of a multi-method implementation assessment strategy for the EMPOWER QUERI, with analyses ongoing. Future use of this method may identify problems not yet described. It remains to be seen whether reflections data directly inform understandings of implementation or patient outcomes once the larger, four-year, multi-site studies (CV Toolkit and CCWV) are complete; however, they have already proven their value as a tool for capturing implementation events and informing problem-solving and sensemaking by implementation team members. The ideal frequency for reflections is unknown and may be project-specific. Because the method is relatively informal and lightly structured, the quality of the resulting data may depend on the training and experience of the individual leading the reflections. Future research should examine whether use of the periodic reflections method is feasible across implementation studies with differing needs, relying upon differing theoretical frameworks, and conducted by project team members with different methodological training.

## Conclusion

Periodic reflections offer a feasible method for incorporating an ethnographically-informed approach into pragmatic implementation, with benefits for allowing observation and documentation of implementation processes and supporting reflection as an activity by the implementation team. Strengths of the method including its low staff burden, minimal cost, ability to be iteratively adapted to meet changing study needs, and utility in supporting observation and documentation of dynamic implementation phenomena over time. Periodic reflections are flexible enough to be compatible with a variety of implementation frameworks or theory-informed approaches [73, 74]. Even so, we have found them to be a useful tool for operationalizing complexity science in implementation [21], and they are perhaps most relevant in the context of frameworks incorporating greater emphasis on multi-level settings, change over time, and ongoing adaptation or process evaluation [10, 13]. They are likely to be of benefit as a component of multi-method evaluation plans accompanying a variety of implementation study designs, with enhanced value for studies occurring across multiple sites.

## Abbreviations

CCWV: Implementation of Tailored Collaborative Care for Women Veterans
CV Toolkit: Facilitating Cardiovascular Risk Screening and Risk Reduction in Women Veterans
DPP: Tailoring VA’s Diabetes Prevention Program to Women Veterans’ Needs
EMPOWER: Enhancing Mental and Physical health of Women through Engagement and Retention
PC-MHI: Primary Care-Mental Health Integration. PI: principal investigator. QI: quality improvement
QUERI: VA Quality Enhancement Research Initiative
REP: Replicating Effective Programs
VA: Department of Veterans Affairs
